# Unveiling the Role and Stabilization Mechanism of Cu^+^ into Defective Ce-MOF Clusters during CO Oxidation

**DOI:** 10.1021/acs.jpclett.4c00324

**Published:** 2024-04-03

**Authors:** Sergio Rojas-Buzo, Davide Salusso, Thanh-Hiep Thi Le, Manuel A. Ortuño, Kirill A. Lomachenko, Silvia Bordiga

**Affiliations:** † Instituto de Tecnología Química, Universitat Politècnica de València - Consejo Superior de Investigaciones Científicas, Av. de los Naranjos, s/n, 46022 Valencia, Spain; ‡ European Synchrotron Radiation Facility, 71 avenue des Martyrs, CS 40220, 38043 Grenoble Cedex 9, France; § Centro Singular de Investigación en Química Biolóxica e Materiais Moleculares (CIQUS), University of Santiago de Compostela, 15782 Santiago de Compostela, Spain; ∥ Department of Chemistry and NIS Centre, University of Turin, Via Giuria 7, 10125 Turin, Italy

## Abstract

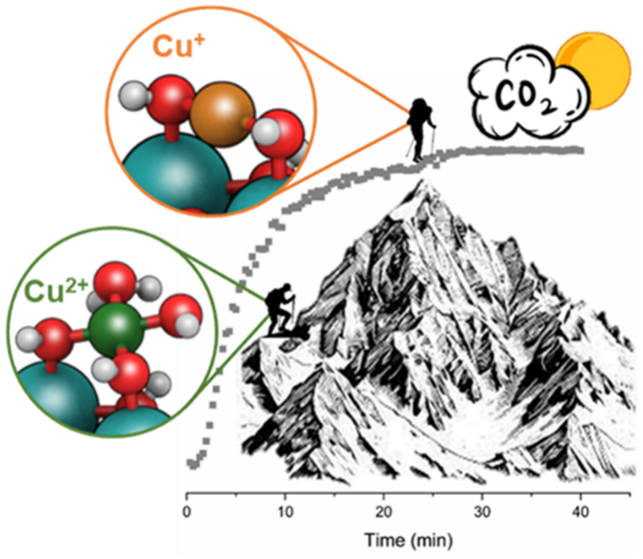

Copper single-site catalysts supported on Zr-based metal–organic frameworks (MOFs) are well-known systems in which the nature of the active sites has been deeply investigated. Conversely, the redox chemistry of the Ce-counterparts is more limited, because of the often-unclear Cu^2+^/Cu^+^ and Ce^4+^/Ce^3+^ pairs behavior. Herein, we studied a novel Cu^2+^ single-site catalyst supported on a defective Ce-MOF, Cu/UiO-67(Ce), as a catalyst for the CO oxidation reaction. Based on a combination of *in situ* DRIFT and *operando* XAS spectroscopies, we established that Cu^+^ sites generated during catalysis play a pivotal role. Moreover, the oxygen vacancies associated with Ce^3+^ sites and presented in the defective Cu/UiO-67(Ce) material are able to activate the O_2_ molecules, closing the catalytic cycle. The results presented in this work open a new route for the design of active and stable single-site catalysts supported on defective Ce-MOFs.

The development and understanding of active species involved in single-site catalysis represent a challenge for a performance-driven rational catalyst design.^1−5^ In this sense, catalytic activity can be attributed to isolated metal sites but also to the action of the support.^6−8^ Conventional supports based on metal oxides are being replaced over time by nanomaterials with a focus on maximizing the reactive surfaces. In this sense, metal–organic frameworks (MOFs), composed of exposed metallic clusters connected by organic ligands, represent potential candidates to stabilize single-site catalysts due to their unique metal–support interactions.^9−11^ In particular, Zr-based MOFs have attracted a lot of attention due to (1) the presence on the nodes of defective OH/OH_2_ groups able to coordinate metal single-sites and (2) their exceptional structural stability during catalysis.^12−15^ Ce-based MOFs constitute an appealing alternative due to their unique electronic properties, for instance, showing accessible nodes containing reduced Ce^3+^ sites.^16,17^ However, this family of materials has not been widely investigated. Recently, we reported a Pt single-site catalyst supported on a Ce-MOF for the CO oxidation reaction.^18^ The presence of accessible Ce^3+^ sites^19^ and, consequently, oxygen vacancies, played an important role during catalysis. In this regard, singly dispersed Cu sites on Ce-MOFs could offer distinctly enhanced performance with respect to the Zr-counterparts. Herein, we prepared for the first time a defective Cu/UiO-67(Ce) material able to stabilize at contemporately Ce^3+^ and Cu^+^ species during real CO oxidation conditions. Cu incorporation into the UiO-67(Ce) nodes via a solvothermal method was performed by using Cu(OAc)_2_·H_2_O as metallic precursor (Figure 1a). The resulting material, denoted as Cu/UiO-67(Ce), retains the crystal structure of the UiO-67 phase, and no evidence of Cu-based nanoparticles, neither metallic nor oxides, were detected in the X-ray diffractogram (Figure S1). Moreover, elemental mapping of Cu was in high accordance with the mapping of those of O and Ce, which indicates that Cu species are homogeneously distributed along the Ce-MOF crystals (Figure S2).

**Figure 1:**
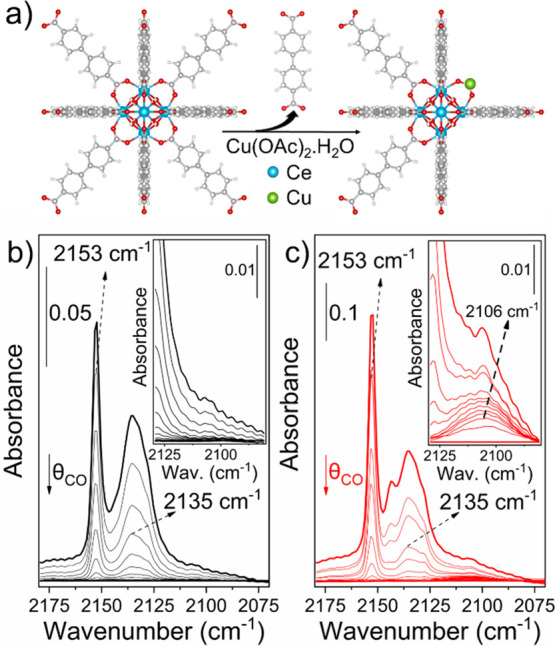
(a) Synthesis of Cu/UiO-67(Ce). Difference IR spectra of CO desorption at LNT on the (b) UiO-67(Ce) and (c) Cu/UiO-67(Ce).

Inductively coupled plasma (ICP) analysis of Cu/UiO-67(Ce) shows the incorporation of ∼2.3 wt % Cu, which corresponds with ∼1 Cu atom per Ce_6_ cluster. UV–vis spectrum of the Cu/UiO-67(Ce) shows an absorption band at ∼13000 cm^–1^, which has been associated with a d-d transition of Cu^2+^ sites (Figure S3).^20^ The Brunauer – Emmett – Teeller (BET) surface area of the as-synthesized Cu/UiO-67(Ce) obtained by the N_2_ physisorption isotherm measured at 77 K was 1740 m^2^·g^–1^ (Table S1 and Figure S4), similar to that obtained for the parent UiO-67(Ce). However, a small hysteresis on the desorption profile suggests that during Cu incorporation some structural defects are formed.^21^ The pore size distribution obtained by applying a DFT model shows the presence of some features in the mesoporous region that were formed by the linker vacancies (inset in Figure S4). This fact was also corroborated by the combination of TGA and ICP analyses (Figure S5 and Table S1, respectively). After the Cu impregnation, the UiO-67(Ce) material increases its linker defectivity in an ∼18% while it shows a higher Ce content. This uncoordination on the Ce clusters generated during the missing linker process may explain the increment of Ce^3+^ sites deduced by the decrease on the band gap from 3.14 to 3.04 eV when comparing UiO-67(Ce) and Cu/UiO-67(Ce), respectively (see inset in Figure S3). From Ce L_3_-edge XANES spectra collected during He activation (section 5 in the Supporting Information), we observed a decrease of the white-line intensity parallel to the rise of a feature at lower energy. This fact suggests the reduction of Ce^4+^ to Ce^3+^ during the thermal activation, in line with our previous reports on UiO(Ce) systems.^22^ Indeed, a linear combination fit (LCF) indicated ∼25% Ce^3+^ formation after activation.

CO adsorption at liquid nitrogen temperature (LNT) was then monitored by IR spectroscopy on both samples, UiO-67(Ce) and Cu/UiO-67(Ce), to evaluate Cu redox behavior. The spectrum of UiO-67(Ce) after being activated at 110 °C under dynamic vacuum (<5.10^–4^ mbar) shows the presence of a band centered at 3648 cm^–1^ that can be assigned to the ν(OH) stretching mode of the (μ_3_–OH)Ce_6_ cluster and two small features at 3635 and 3642 cm^–1^, associated with defective OH/OH_2_ groups (Figure S6). After Cu incorporation, the μ_3_–OH groups remained nearly untouched, suggesting that Cu anchoring occurs on defective Ce sites (Scheme S1). Then, we measured CO adsorption at nominal 100 K on UiO-67(Ce) (Figure 1b). The bands at 2153 and 2135 cm^–1^ are associated with CO interaction with hydroxyl groups and physisorbed CO, respectively.^22,23^ Conversely, in case of Cu/UiO-67(Ce), we clearly noticed that while the signals related with OH sites and CO liquid-like quickly disappeared during the CO desorption, a small contribution at 2106 cm^–1^, related to CO-Cu^+^ adduct,^24^ persisted (Figure 1c). To corroborate the chemical nature of this interaction, we treated Cu/UiO-67(Ce) with H_2_ at 200 °C to favor the Cu reduction. As can be observed in Figure S7, the intensity of the band at 2106 cm^–1^ increased considerably after the reduction treatment, confirming its assignment to CO-Cu^+^ moieties.

To approach realistic reaction conditions, we carried out an *in situ* IR experiment at 200 °C, in which the Cu/UiO-67(Ce) material was exposed to four different chemical environments (Figure S8). (a) During helium activation, the material lost the DMF molecules trapped in the MOF pores, while the μ_3_–OH groups remained nearly untouched. (b) During H_2_ treatment, the band at 3574 cm^–1^, related to OH stretching modes in Cu–OH sites,^25^ was consumed at the same time that some water molecules were generated. This can be explained if we consider partial reduction of Cu and Ce. Moreover, the crystalline nature of the material is maintained after this treatment (see inset in Figure S8b). (c) The Cu/UiO-67(Ce) was then exposed to an aerobic atmosphere to reoxidize its surface. (d) Finally, CO (10% in He) was flowed at 200 °C into the cell. Interestingly, we observed a contribution at ∼2110 cm^–1^ (see inset in Figure S8d), assigned previously to CO-Cu^+^ adduct, which indicates the CO interaction even at higher temperatures.

We next examined the catalytic behavior of Cu/UiO-67(Ce) for CO oxidation in a plug-flow reactor with a reaction mixture (GHSV = 11250 mL/g_cat_·h, 6.67% CO, and 3.33% O_2_) at 200 °C for 40 h (Figure 2a). First, the catalyst passed by an activation period of ∼25 h, until it reached 95% CO conversion. After that, its catalytic performance was maintained for 15 h without any evidence of deactivation. A blank experiment was carried out by using UiO-67(Ce) as a catalyst. The calculated catalytic activity at 200 °C was ∼60 times lower, underlying the key role of Cu during catalysis (Figure S9). Moreover, the material stability was also checked at lower temperatures (125 °C), and conversion values of ∼16% remained after 17 h.

**Figure 2:**
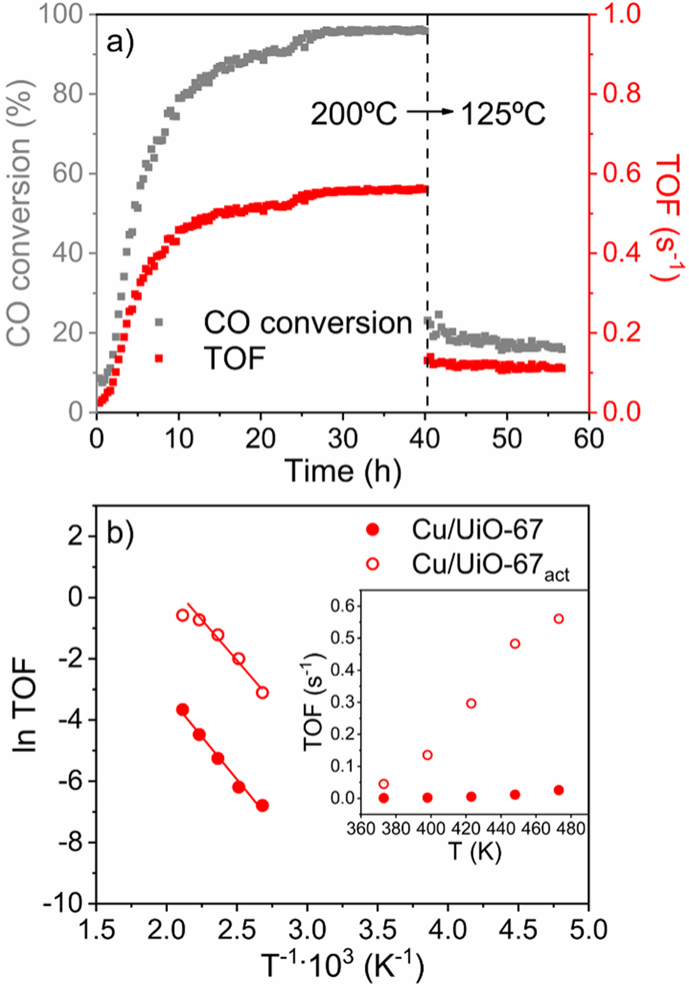
(a) Long time on stream (TOS) curves based on the CO conversion (gray squares) and calculated TOFs (red squares) for the Cu/UiO-67(Ce) at 200 and 125 °C. (b) Arrhenius plots for Cu/UiO-67(Ce) (filled red circles) and Cu/UiO-67(Ce)_act_ (empty red circles). The determination of the activation energy was made within 100–175 °C range where lower CO conversion values were attained. Calculated TOFs at different temperatures are reported in the inset.

Finally, the CO_2_ production normalized by the amount of copper at different temperatures was calculated for the activated catalyst, denoted as Cu/UiO-67(Ce)_act_. The obtained TOF values were considerably higher for this material than for the fresh catalyst (see the inset in Figure 2b). Moreover, the calculated activation energies within the 100–175 °C range for Cu/UiO-67(Ce)_act_ and Cu/UiO-67(Ce) were 44.2 and 46.5 kJ/mol, respectively. Under low-temperature regimes, the Cu/UiO-67(Ce)_act_ presented considerably improved TOF values for the CO oxidation reaction compared to representative examples in the literature of Cu catalysts supported on Ce-/Zr-based materials, including CeO_2_ and Ce-/Zr-MOFs, with different Cu/Ce/Zr ratios (Table S2).^17,26−29^

Even if the crystallinity and the surface area decreased (Table S3 and Figure S10), the phase purity of the UiO-67 structure together with the catalytic activity were maintained after 40 h under a CO oxidation stream at 200 °C (Figure S11). Moreover, no evidence of Cu aggregation was found in the diffractogram. CO adsorption at LNT followed by IR was employed to unravel the redox nature of Cu sites after catalysis. As can be deduced from Figure S12, an increment in the proportion of Cu^+^ sites after the catalytic test was detected during CO desorption, which could explain the higher catalytic activity of the material obtained after the CO oxidation stream.

To determine Cu and Ce oxidation states under real reaction conditions, we employed *in situ* and *operando* DRIFT and XAS spectroscopies. DRIFT (Diffuse Reflectance IR Fourier Transform) spectra collected during 12 h of reaction at 200 °C under stoichiometric CO/O_2_ conditions show two main aspects to be considered (Figure 3a): (1) the appearance of roto-vibrational stretching modes related with gaseous CO_2_ phase (see left inset in Figure 3a) and (2) the increasing of the ∼2110 cm^–1^ band intensity, related to CO adsorbed on Cu^+^ sites (see right inset in Figure 3a). Interestingly, the evolution of the CO_2_ gas phase follows the trend of the band at ∼2110 cm^–1^, indicating that the CO oxidation rate increases with the increasing of the Cu^+^ concentration.

**Figure 3:**
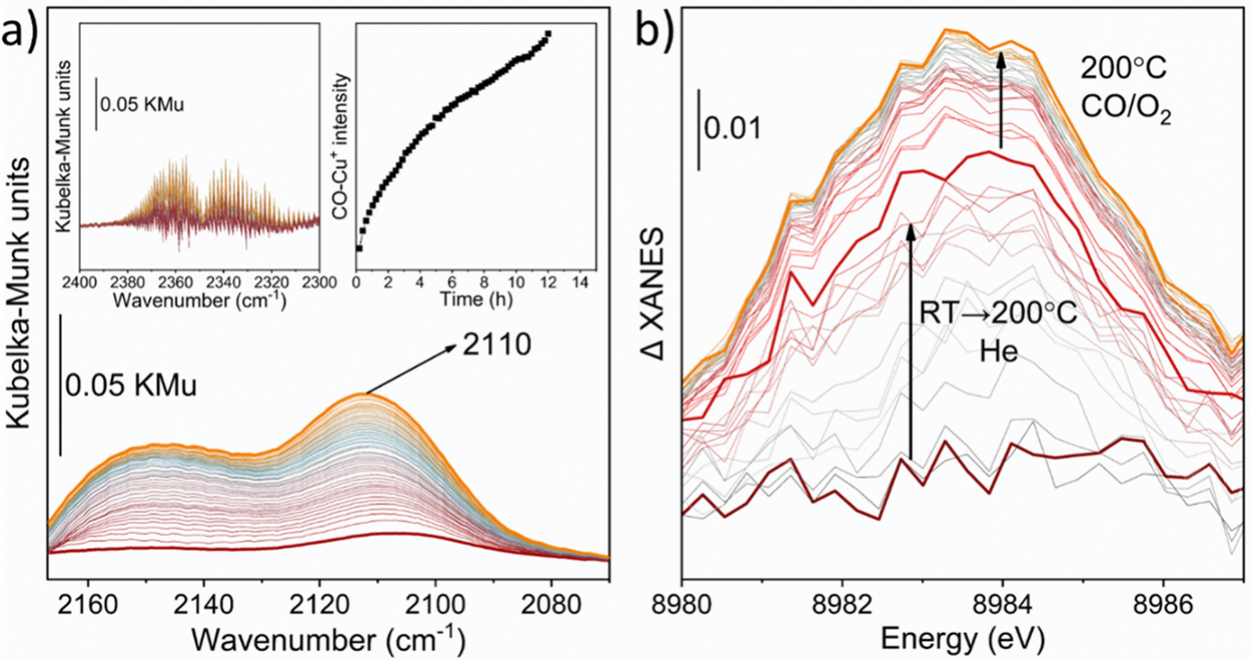
(a) DRIFT spectra collected during CO oxidation at atmospheric pressure and 200 °C for 12 h. Time evolution was described from a dark red to orange line. CO_2_ gas-phase and 2110 cm^–1^ band intensity evolution are described in the left and right insets, respectively. (b) Detail of Cu^+^ ΔXANES operando Cu K-edge spectra collected during activation (dark red to red line) and the CO oxidation reaction (red to orange line).

To further investigate the Ce and Cu nature, we have monitored Cu K- and Ce L_3_-edges XANES spectra during the CO oxidation reaction at BM23 beamline of the ESRF.^30^ Cu K-edge XANES spectrum of as-prepared Cu/UiO-67(Ce) (Figure S14a) indicated Cu mostly presented as Cu^2+^ with a highly hydrated local environment.^31^ Fourier transform analysis of the EXAFS part indicated the presence of an intense Cu–O single scattering path while the Cu second coordination shell presented only weak oscillations suggesting the presence of isolated sites (Figure S14b,c). During thermal activation a minor contribution around 8984 eV was observed in the spectra pre-edge (see ΔXANES Figures 3b and S15) which can be ascribed to a Cu^+^ 1s → 4p transition.^29,32−35^

We next performed DFT simulations^29,36,37^ to further support the XAS data. We employed the M06-L density functional^38^ in Gaussian 16,^39^ and the computational data is freely available in the ioChem-BD platform.^40^ We first prepared a neutral finite-size cluster of as-synthesized UiO-67(Ce).^41^ To create a vacancy, we removed one linker and capped the unsaturated Ce atoms with OH/OH_2_; then, we include a hydrated mononuclear Cu^2+^ species (doublet spin state) in different positions of the node (see Scheme S1). We found four relevant geometries (**Cu-1**–**4**, Figure S13), and Figure 4 shows one of them (**Cu-2**) as an example. The Cu atom presented a slightly distorted square-planar environment (Cu–O bond distances of 1.9–2.0 Å) and a H_2_O molecule in apical position (Cu···O distance of 2.362 Å).

**Figure 4:**
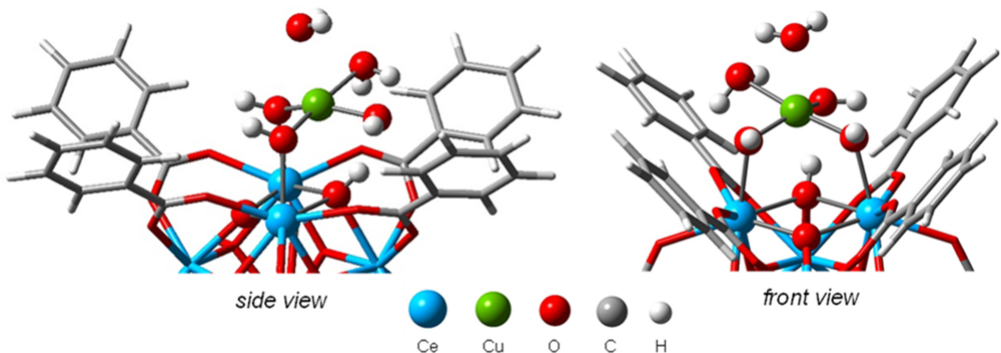
DFT-optimized structure of **Cu-2** of the as-synthesized Cu/UiO-67(Ce).

The FT-EXAFS fit performed using the above-mentioned structures resulted in the same outcome (Figures S16 and S17 and Tables S4 and S5), indicating the presence of 4 surrounding oxygens in the first coordination shell located at 1.87 ± 0.04, 1.93 ± 0.03 (two equidistant), and 1.98 ± 0.06 Å. Nevertheless, only structures **Cu-2** and **Cu-4** presented H_2_O in the apical position, which is in line with the observed intense XANES white line. During >20 h of reaction, Cu^+^ content increased (Figures 3b and S18a) while the CO_2_ signal increased after 10 h (Figure S18d). It is noteworthy that after 24 h of reaction, the EXAFS spectra did not present significant changes, indicating high Cu stability on the Ce nodes (Figure S18b,c). The same *operando* experiment was repeated monitoring Ce L_3_-edge, which indicated the presence of Ce^4+^ in the as-prepared sample and an increase of Ce^3+^ content during thermal activation (Figure S19a) up to ∼25% as quantified by LCF (Figure S19b). Under the CO oxidation condition, we observed an increase of CO consumption while the spectra after 24 h presented a slightly higher content of Ce^3+^. It is noteworthy as the CO_2_ signal presented more noise due to the lower amount of catalyst employed to work in transmission XAS mode at the Ce L_3_-edge, causing an overall lower CO conversion.

In summary, we have designed a Cu single-site catalyst supported on a Ce-MOF with enhanced activity for the CO oxidation reaction. *In situ* and *operando* spectroscopies reveal the simultaneous presence of (1) Ce^3+^ and (2) Cu^+^ sites on the defective Ce-nodes during the reaction. Moreover, the defect-engineered Cu/UiO-67(Ce), prepared in this work, is one of the most active single-site/MOF catalysts based on Earth-abundant elements for the CO oxidation. These results open new perspectives for the design of single-site catalysts stabilized on Ce-based MOFs.

## Supporting Information Available

Synthesis and characterization of the materials (PXRD patterns, FESEM/EDX analysis, UV–vis spectra, N_2_ adsorption–desorption isotherms, TGA, FTIR spectra). Catalytic testing (Catalytic evaluation, comparison of the catalytic performance with other Cu catalysts supported on Ce-based materials, characterization of the material after catalysis). Computational details. XAS spectroscopy (Cu K-edge, FT-EXAFS fit, operando Ce L_3_-edge) (PDF)
