# Supplementary material for: Unveiling the Role and Stabilization Mechanism of Cu+ into Defective Ce-MOF Clusters during CO Oxidation
Source: J Phys Chem Lett. 2024 Apr 3;15(14):3962–7. doi: 10.1021/acs.jpclett.4c00324 (PMC11017307; doi:10.1021/acs.jpclett.4c00324)
Supplement: Supplementary file 1 — jz4c00324_si_001.pdf [file jz4c00324_si_001.pdf]

Supplementary Information

Unveiling the Role and Stabilization Mechanism of Cu^+ into Defective Ce-MOF Clusters during CO Oxidation

Sergio Rojas-Buzo,[1]* Davide Salusso,[2] Thanh-Hiep Thi Le,[3] Manuel A. Ortuño,[3] Kirill A. Lomachenko[2] and Silvia Bordiga[4]*

[1]Instituto de Tecnología Química, Universitat Politècnica de València - Consejo Superior de Investigaciones Científicas, Av. de los Naranjos, s/n, 46022 Valencia, Spain

[2]European Synchrotron Radiation Facility, 71 avenue des Martyrs, CS 40220, 38043 Grenoble Cedex 9, France.

[3]Centro Singular de Investigación en Química Biolóxica e Materiais Moleculares (CIQUS), University of Santiago de Compostela, 15782 Santiago de Compostela, Spain.

[4]Department of Chemistry and NIS Centre, University of Turin, Via Giuria 7, 10125 Turin, Italy.

Table of contents

1. Sample Preparation 2

1.1. Synthesis of UiO-67(Ce) 2

1.2. Synthesis of Cu/UiO-67(Ce). 2

2. Sample Characterization 2

2.1. Powder X-ray diffraction (PXRD) patterns 6

2.2. Field Emission Electron Microscopy (FESEM) image and EDX maps of Ce, Cu and O 6

2.3. UV-vis analysis 7

2.4. N_2 adsorption-desorption isotherms 8

2.5. Thermogravimetric analysis (TGA) 9

2.6. IR characterization 10

3. CO oxidation reaction. 13

3.1. Cu effect on the catalytic performance of the UiO-67(Ce) material 13

3.2. Comparison of the catalytic performance for the Cu/UiO-67(Ce) with other state-of-the-art Cu supported on different Ce-based materials 14

3.3. Cu/UiO-67(Ce) characterization after catalysis 15

4. Computational Details 17

5. Operando X-Ray Absorption Spectroscopy (XAS) 19

5.1. Cu K-edge 19

5.2. FT-EXAFS fit procedure 21

5.3. Operando Ce L_3-edge 26

6. References 27

1. Sample Preparation

1.1. Synthesis of UiO-67(Ce).

The synthesis has been carried out following a scaled-up previously reported recipe[1]: H_2BPDC (Biphenyl-4,4´-dicarboxylic acid) (1.42 g, 5.86 mmol) and 33 mL of *N,N*-dimethylformamide (DMF) were placed in a round-bottomed flask. Then, an aqueous solution of cerium ammonium nitrate (11 mL, 0.53 M) was added to the mixture. The flask with the resulting solution was sealed and heated under magnetic stirring for 15 min at 100°C. The as-obtained yellow precipitate was decanted by centrifugation from the mother solution and washed with dimethyl sulfoxide (DMSO), DMF and acetone. To avoid the presence of unreacted organic ligand on the MOF pores, the material was treated overnight with DMF at 100ºC. Finally, the UiO-67(Ce) was decanted and washed with DMF and acetone, and subsequently dried in an oven at 100ºC for 1 hour.

1.2. Synthesis of Cu/UiO-67(Ce).

The Cu-supported UiO-67(Ce) material was prepared following a previous synthetic method with some modifications.[2] Firstly, $Cu(OAc)_2{\cdot}H_2O$ (70 mg, 0.35 mmol) was dissolved in 67 mL of DMF. The resulting solution was added to a Teflon-lined autoclave containing 1.25 g of UiO-67(Ce). The suspension was heated at 100°C in an oven during 24 h. The as-synthesized Cu/UiO-67(Ce) was decanted and washed with DMF and acetone. Finally, the material was dried in an oven at 100ºC for 1 hour.

2. Sample Characterization

The PXRD measurements were carried out using the Bragg–Brentano geometry with a PANalytical PW3050/60 X'Pert PRO MPD diffractometer with a Cu anode (Kα = 1.5418 Å) and an X'Celerator detector.

The morphology of the samples, placed on a carbon tape, was studied by field emission scanning electron microscopy (FESEM) using a FEG-SEM TESCAN S9000G microscope, equipped with a FEG, Scottky type of source. Energy

dispersive X-ray (EDX) spectrometry analyses were performed at the same time to obtain the metallic distribution on the MOF particles.

DR UV–vis spectra were measured with a Varian Cary5000 spectrophotometer, equipped with a diffuse reflectance sphere, where the samples were placed in powder form. The spectra were collected in a reflectance mode and successively converted as Kubelka–Munk F(R) function.

Isothermal N_2 physisorption measurements at liquid nitrogen temperature (LNT) were performed on a Micromeritics 3Flex. Prior to the measurement, the powders were degassed 4h at 110°C. Specific surface areas using the Brunauer-Emmett-Teller (BET) model were calculated following the Rouquerol´s criterion. Pore size distribution was obtained by applying the N_2-Cylindrical Pores-Oxide Surface DFT model.

Thermogravimetric analysis (TGA) data were recorded with a TA Instruments Q600 thermobalance in air flow (100 mL/min) with a ramp of 5°C/min from room temperature (RT) to 500°C working with ~5 mg of sample in an alumina crucible.

ICP analyses were carried out in a Varian 715-ES ICP-Optical Emission spectrometer after solid dissolution in H_2SO_4/H_2O_2 aqueous solution.

Fourier Transform Infrared (FT-IR) spectroscopy in transmission mode was employed to characterize surface properties of the materials by following the adsorption/desorption of CO as probe molecule. Absorption/transmission FTIR spectra were collected using a Bruker Vertex 70 spectrophotometer equipped with a Mercury Cadmium Telluride (MCT) cryo-detector in the 4000-600 cm^{-1} range with 2 cm^{-1} resolution. Powders were pressed in self-supporting discs (~4 mg/cm^2) and placed in quartz IR cells suitable for thermal treatments in controlled atmosphere and for spectra recording even at LNT. Before IR measurements, catalysts were activated from RT to 110°C at 5°C/min holding at 110°C until the pressure reached 5.10-4 mbar. The pre-reduced Cu/UiO-67(Ce) was treated as follow: 1) The sample was degassed at 110°C (5°C/min) until dynamic vacuum. 2) Then, H_2 (100 mbar) was dosed into the cell and it was heated up to 200°C during 30 min. 3) Finally, the

cell was cooled down and evacuated until the pressure reached 5.10-4 mbar. Spectra were treated using Bruker OPUS spectroscopy software. All the reported spectra were normalized for the pellet weight and area.

In situ IR experiments were conducted with a Bruker Invenio spectrophotometer equipped with a 'Sandwich type' IR cell.[3] The catalyst wafer was inserted in the round-shaped homemade sample holder. Bottles of gases were connected to Bronkhorst EL-flow mass flowmeters. Background spectra was collected after flushing the empty cell for 30 minutes under He flow (30 mL/min). Spectra were recorded with 60 kHz and every spectrum was the results of the average of 32 scans (ca. 60 s/scan).

The measurement protocol consisted in: 1) catalyst activation at 200°C (3°C/min) under He flow (30 mL/min) for 30 min. 2) Sample reduction at 200ºC under a pure H_2 flow (30 mL/min) during 30 min to be then removed with pure He during 10 min. 3) This process was then repeated but O_2 was used instead H_2. 4) After the last cleaning step in He, the pellet was flushed with CO (30 mL/min, 3% CO and 27% He) at 200ºC during 30 min.

DRIFT experiments were conducted with a Bruker Invenio spectrophotometer equipped with a commercial cell (PIKE TECHNOLOGIES, DIFFUSIR™). Measurements were performed under similar reaction conditions followed in the catalytic tests: the Cu/UiO-67(Ce) was activated at 200ºC during 30 minutes passing a flow of He (45 mL/min). Then, the reaction mixture was flowed (45 mL/min, containing 2.22% CO and 1.11% O_2) at atmospheric pressure at 200ºC for 12 h. Spectra were collected (every 10 min) and reported in Kubelka-Munk units. Background subtraction of the spectra was performed using a spectrum recorded initially in a flow of He (45 mL/min) at 200ºC.

X-Ray Absorption Spectra (XAS) were measured at BM23 beamline of the European Synchrotron Radiation Facility (ESRF) during in-house beamtimes IH-CH-1712 and IH-CH-1726.[4]The storage ring was operating in 16 bunch mode with maximum ring current of 75 mA for Cu K-edge experiment while Ce L_3-edge were collected in 4 bunch mode with maximum ring current of 32 mA. Cu K-edge and Ce L_3-edge XAS

spectra were collected in transmission mode on mass optimized pellets of ≈0.2 cm^2 and 6.8 and 1.8 mg, respectively. Two ion chambers (IC) were employed to measure incoming beam (IC_0) and beam transmitted from the sample (IC_1). A third IC (IC_2) was located after IC_1 to measure transmitted beam from a reference sample employed for energy alignment purposes. Cu K-edge spectra were collected in the 8.8 – 10.2 keV energy range in continuous mode with 0.3 eV/point energy resolution and 0.04 s/point integration time for a total of 3.5 minutes/scan. Cu metal foil was used for energy calibration and alignment. Ce L_3-edge spectra were collected in the 5.55 – 6.15 keV energy range with 0.25 eV/point energy resolution and 0.04 s/point integration time for a total of 2 minutes/scan. Pellet of CeO_2 was used for energy alignment. Spectra of steady states resulted from the average of 10 spectra. Spectra were energy aligned, background subtracted and edge jump normalized with Larch based python script.[5] All the reported FT-EXAFS spectra were extracted with Hanning window in the 2.2–11.4 $Å^{-1}$ k-range.

The sample was placed in a home-made reaction cell suitable for thermal treatments under gas flows (Transmission Pellet Cell, TPC). Inlet gas flows were provided by Bronkhorst mass flow controllers while reactants consumption/products formation were determined with an online Mass Spectrometer connected to the cell outlet. The operando XAS experiment followed a protocol similar to the one employed for DRIFT experiment *i.e.*: the sample was heated to 200ºC (5ºC/min) under He flow (45 mL/min) and kept at this temperature for 30 minutes. Then, the reaction mixture was flowed (45 mL/min, containing 4.44% CO and 2.22% O_2) at atmospheric pressure at 200ºC for 20 h. The cell was then purged under He (50 mL/min) for 30 minutes and cooled to RT (5ºC/min). The outlet gas composition was monitored with a mass spectrometer.

Spectra of CuO, Cu_2O, CeO_2 and $Ce(NO_3)_3$ references were measured on mass optimized pellets in transmission mode with the same parameters. $Cu(OAc)_2$ spectra was recorded in water solution.

2.1. Powder X-ray diffraction (PXRD) patterns

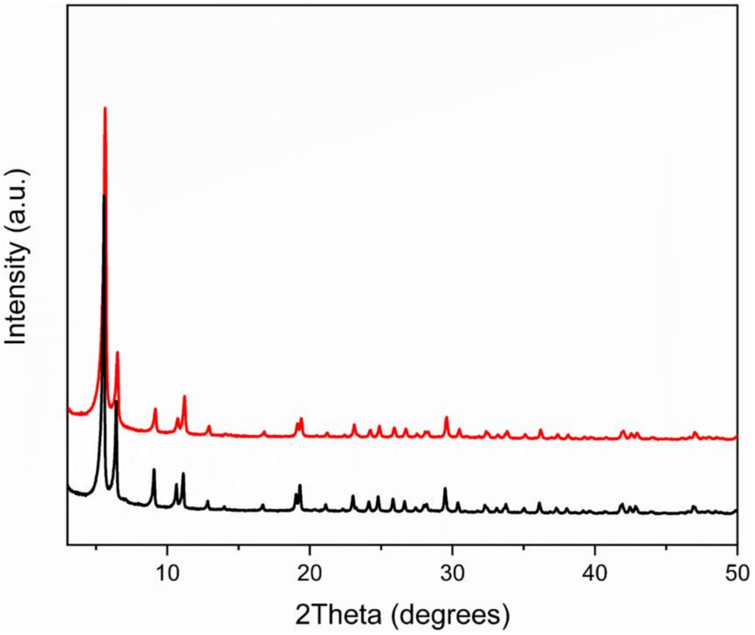

Figure S1. PXRD patterns of the as-synthesized UiO-67(Ce) (black line) and Cu/UiO-67(Ce) (red line).

2.2. Field Emission Electron Microscopy (FESEM) image and EDX maps of Ce, Cu and O

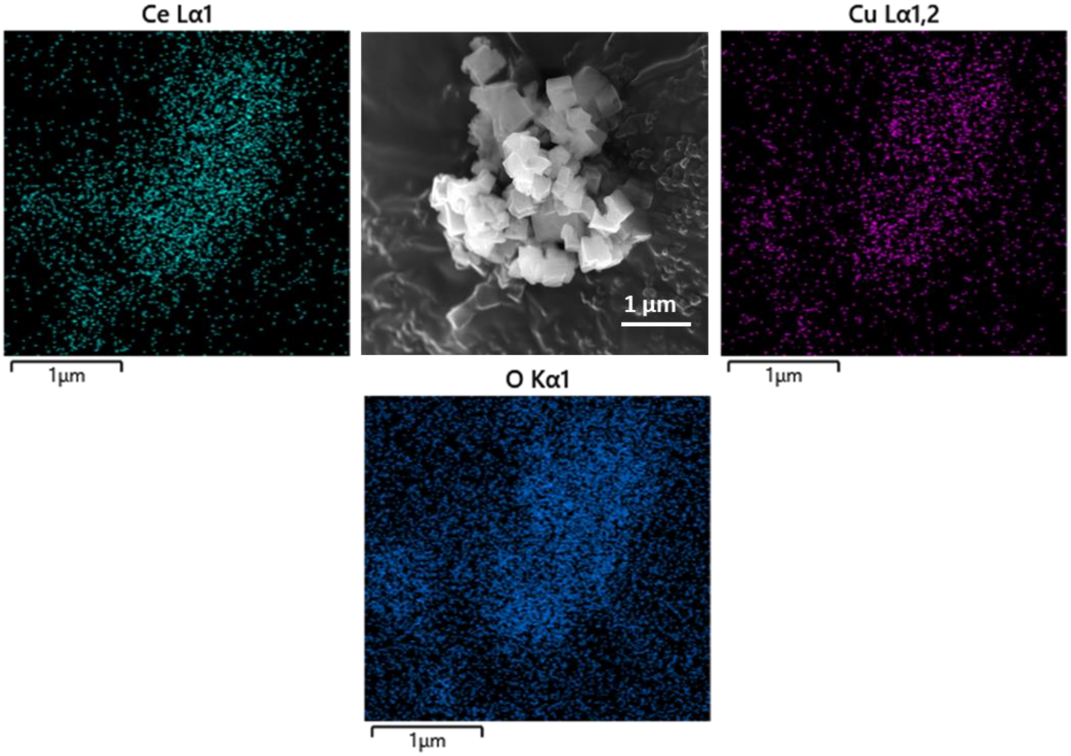

Figure S2. FESEM/EDX analysis of the Cu/UiO-67(Ce).

2.3. UV-vis analysis

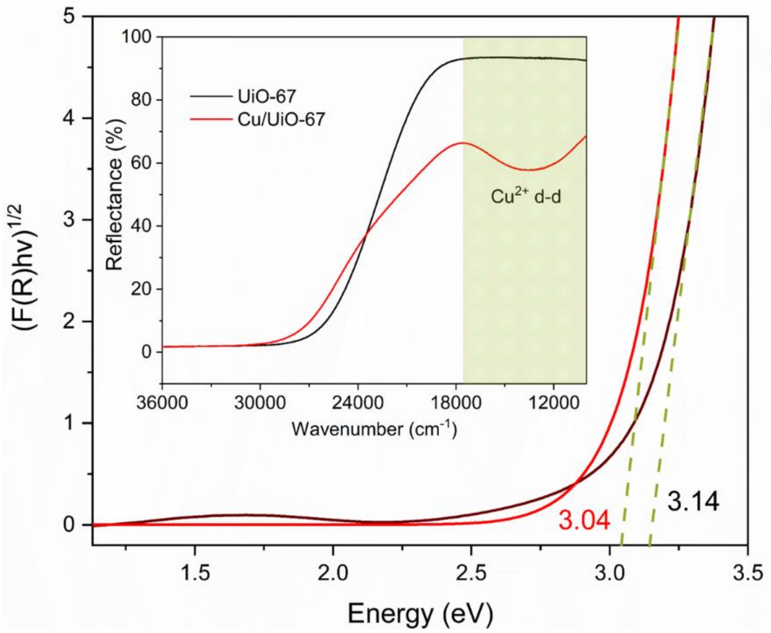

Figure S3. Tauc plots of the UiO-67(Ce) (black line) and Cu/UiO-67(Ce) (red line). DR UV-vis spectra of UiO-67(Ce) (black line) and Cu/UiO-67(Ce) (red line) are reported in the inset.

2.4. N_2 adsorption-desorption isotherms

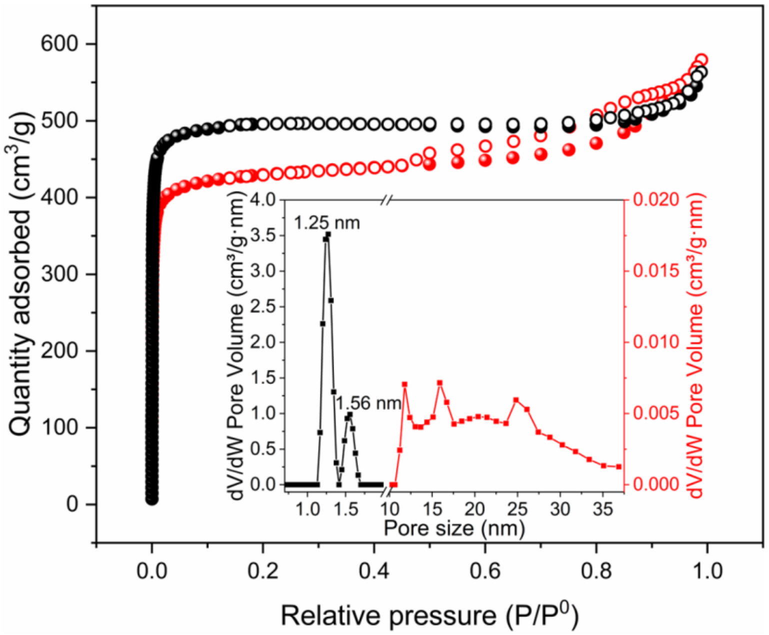

Figure S4. N_2 adsorption-desorption (filled and empty circles, respectively) isotherms of UiO-67(Ce) (black circles) and Cu/UiO-67(Ce) (red circles). Pore size distribution of Cu/UiO-67(Ce) is reported in the inset.

Table S1. Physicochemical properties of the UiO-67(Ce) and Cu/UiO-67(Ce) materials.

Sample	BET surf. area (m^2/g)	Micro. area (m^2/g)	Micro. volume (cm^3/g)	Total pore volume (cm^3/g)	Ce* (wt %)	Cu* (wt %)
UiO-67(Ce)	2045	1935	0.75	0.75	22.4	-
Cu/UiO-67(Ce)	1740	1566	0.65	0.75	26.4	2.3

*Determined by ICP analysis

2.5. Thermogravimetric analysis (TGA)

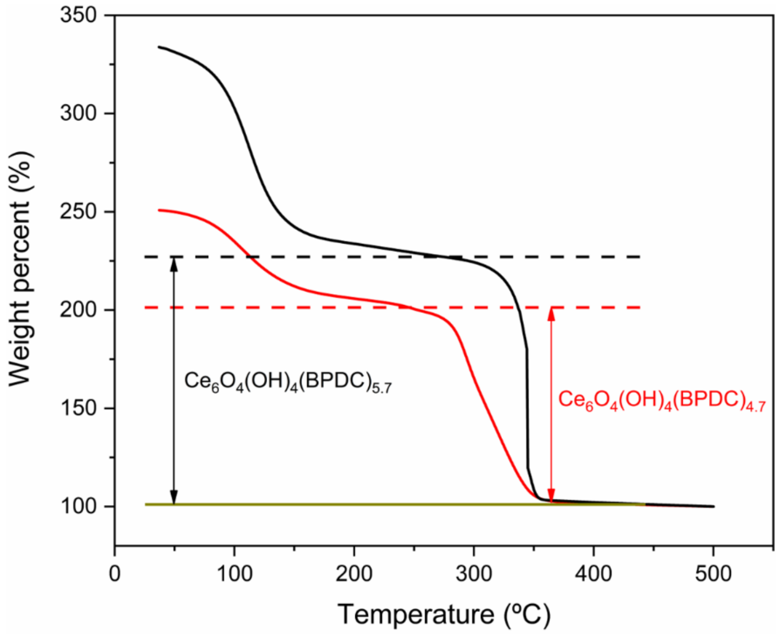

Figure S5. Normalized TGA profiles (recorded under aerobic conditions) of the as-synthesized UiO-67(Ce) (black line) and Cu/UiO-67(Ce) (red line).

2.6. IR characterization

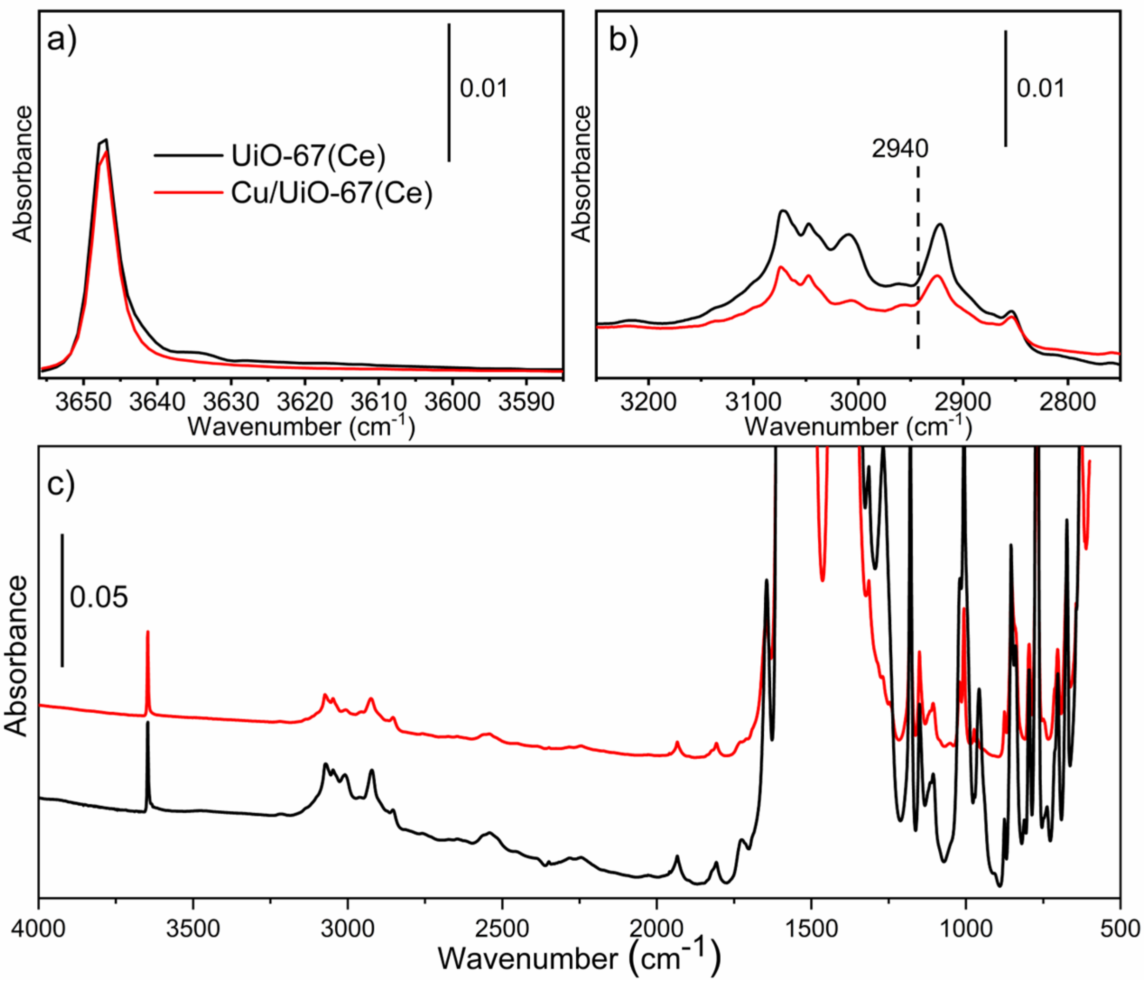

Figure S6. IR spectra of the UiO-67(Ce) (black line) and Cu/UiO-67(Ce) (red line). *v*(OH) and v(CH_3) regions are reported in panel a and b, respectively, while panel c shows the full spectra. The presence of isolated acetates could be discarded since v(CH_3)$_{sym}$ vibrations at ~2940 cm^{-1} in the IR spectrum of Cu/UiO-67(Ce) reported in panel b were not detected.

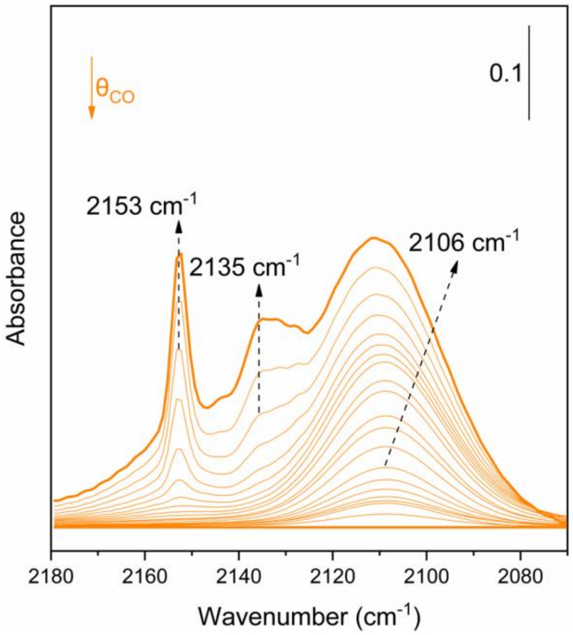

Figure S7. Difference IR spectra of CO desorption at LNT on the pre-reduced Cu/UiO-67(Ce) sample.

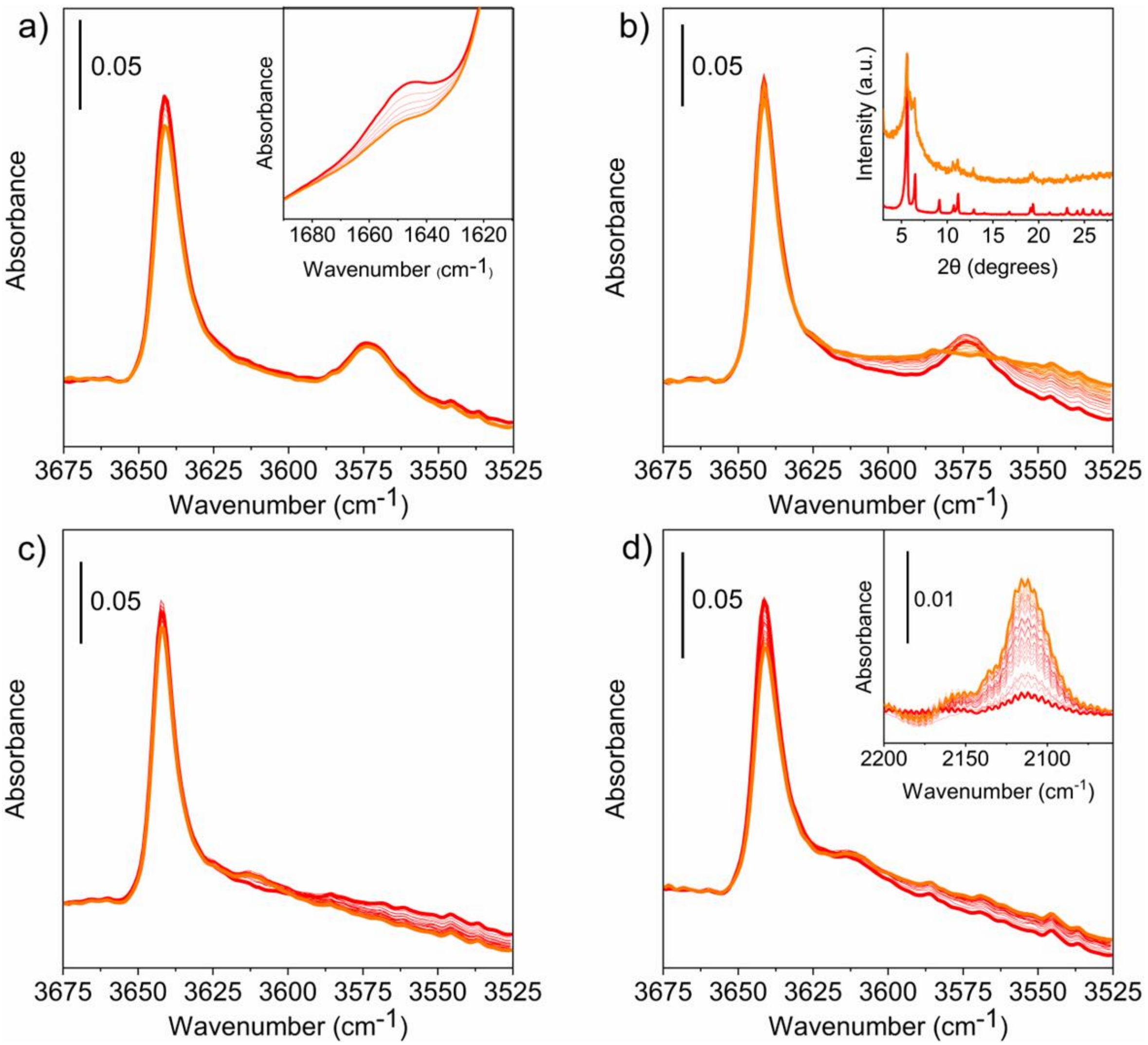

Figure S8. IR spectra of Cu/UiO-67(Ce), focused in the *v*(OH) range, collected a) during He activation (from RT to 200ºC with a 3ºC/min ramp) followed by b) H_2 treatment, c) O_2 treatment and d) CO treatment (10% in He) at 200ºC (time evolution from red to orange line). Inset in panel a describes the desorption of the DMF molecules trapped into the MOF pores while inset in panel d shows the difference IR spectra during CO adsorption focused on *v*(CO) region. PXRD patterns of the fresh Cu/UiO-67(Ce) (red line) and after be treated with H_2 (orange line) are reported in the inset of panel b.

3. CO oxidation reaction.

In a typical experiment, 80 mg of Cu/UiO-67(Ce) were loaded in a conventional tubular plug-flow reactor (ID = 4 mm). The reactor was heated at 200ºC during 30 minutes flowing He through the catalyst (15 mL/min) to eliminate the physisorbed molecules. Then, the reaction mixture was flowed at atmospheric pressure at different temperatures from 100 to 200ºC every 25ºC: 15 mL/min (GHSV = 11250 mL/g_{cat}·h), containing 6.67% CO and 3.33% O_2. The downstream reaction effluents were analyzed continuously by gas chromatography.

3.1. Cu effect on the catalytic performance of the UiO-67(Ce) material

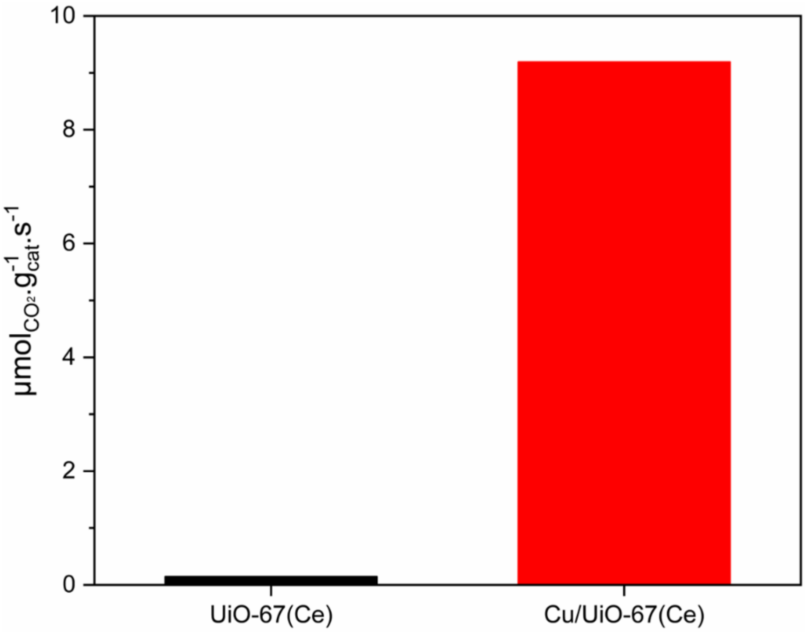

Figure S9. Reaction rates (μmol$_{CO2}$.g^{-1}.s^{-1}) obtained for the CO oxidation using UiO-67(Ce) (black bar) and Cu/UiO-67(Ce) (red bar) as catalysts. (Reaction conditions: GHSV = 11250 mL/g_{cat}·h, 6.67% CO and 3.33% O_2, 200°C).

3.2. Comparison of the catalytic performance for the Cu/UiO-67(Ce) with other state-of-the-art Cu supported on different Ce-based materials

Table S2. Catalytic performance of Cu-catalysts supported on different Ce-based materials.

**Catalyst**	**CO Oxidation Conditions**	**$Cu_{1-x}Ce_x$**	**T (ºC)**	**TOF x 10^{-3} (s^{-1})**	**References**
Cu/UiO-67(Ce)_fresh	15 mL/min (6.67% CO, 3.33% O_2) 11250 mL/g_{cat}·h	$Cu_{0.16}Ce_{0.84}$	125	2.05	This work
Cu/UiO-67(Ce)_act	15 mL/min (6.67% CO, 3.33% O_2) 11250 mL/g_{cat}·h	$Cu_{0.16}Ce_{0.84}$	100	44.89	This work
Cu/UiO-67(Ce)_act	15 mL/min (6.67% CO, 3.33% O_2) 11250 mL/g_{cat}·h	$Cu_{0.16}Ce_{0.84}$	125	135.54	This work
Cu/MOF-808(Ce)	32.5 mL/min (1.0% CO, 2.5% O_2) 78000 mL/g_{cat}·h	$Cu_{0.40}Ce_{0.60}$	125	0.01	[2]
Cu/UiO-66(Zr)*	30 mL/min (1.0% CO, 1.0% O_2) 15000 mL/g_{cat}·h	$Cu_{0.12}Zr_{0.88}$	80	0.22	[6,7]
$CuO-CeO_2$	26 mL/min (1.0% CO, 2.5% O_2) 78000 mL/g_{cat}·h	$Cu_{0.08}Ce_{0.92}$	120	70	[8]
$CuO-CeO_2$	40 mL/min (1.0% CO, 1.0% O_2) 9600 mL/g_{cat}·h	$Cu_{0.10}Ce_{0.90}$	80	34.22	[9]

*Zr instead Ce.

3.3. Cu/UiO-67(Ce) characterization after catalysis

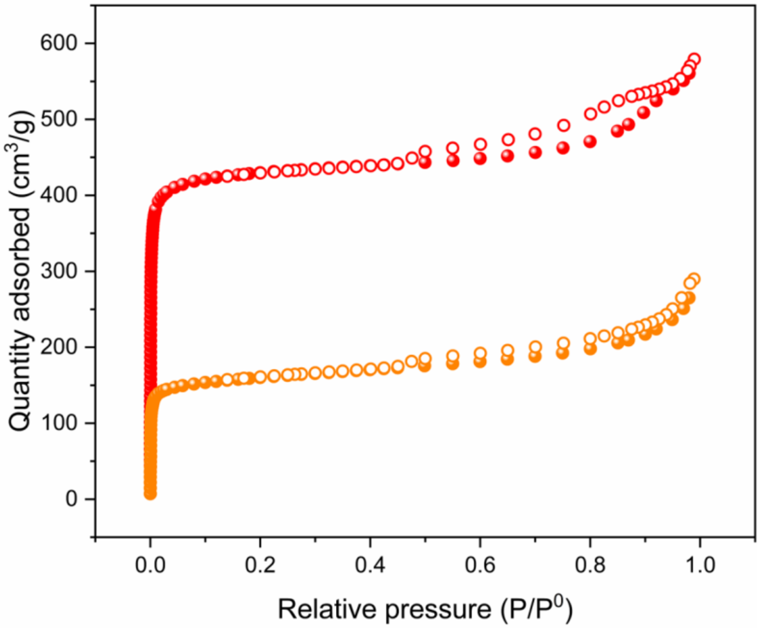

Figure S10. N_2 adsorption-desorption (filled and empty circles, respectively) isotherms of the fresh Cu/UiO-67(Ce) (red circles) and after be tested in the CO oxidation reaction (orange circles).

Table S3. N_2 adsorption isotherm report.

Sample	BET surf. area (m^2/g)	Micro. area (m^2/g)	Micro. volume (cm^3/g)
UiO-67(Ce)	2045	1935	0.72
Cu/UiO-67(Ce)	1740	1566	0.59
Cu/UiO-67(Ce) used	620	475	0.18

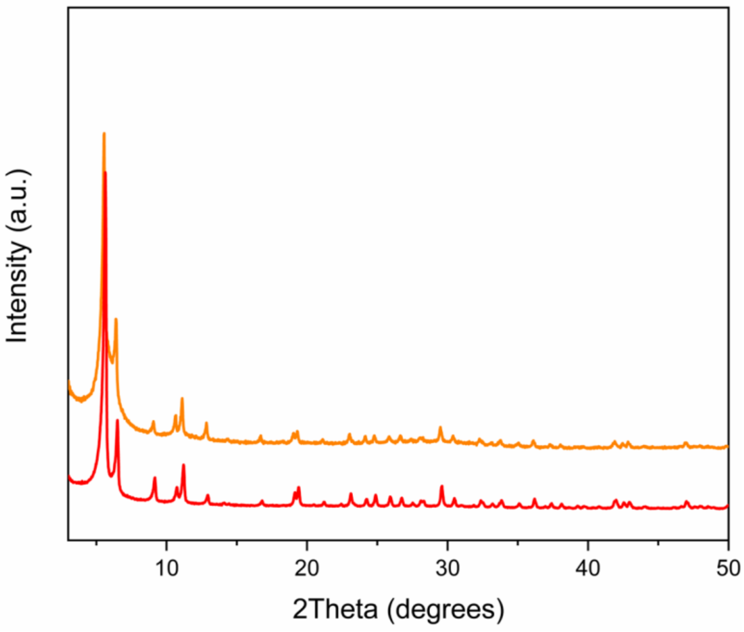

Figure S11. PXRD patterns of the fresh Cu/UiO-67(Ce) (red line) and after be used in the CO oxidation reaction (orange line).

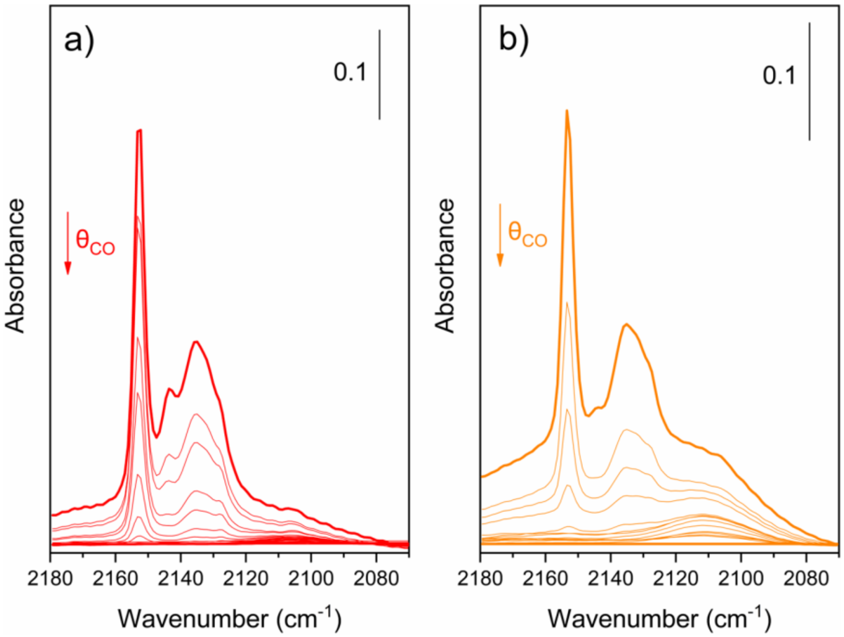

Figure S12. Difference IR spectra of CO desorption at LNT on the a) fresh Cu/UiO-67(Ce) (red line) and b) after the catalytic test (orange line).

4. Computational Details

DFT calculations were carried out with the M06-L density functional[10] as implemented in Gaussian 16.[11] Cu and Ce atoms were described with SDD pseudopotential and its associate basis set,[12] while O, C, and H were described with 6-31G(d,p) basis set.[13–15] Geometry optimizations were performed in the gas phase and frequency calculations confirmed that the optimized structures were minima. Inputs and outputs are available in the ioChem-BD repository[16] through the following database.[17]

Simulations were performed on a finite-size cluster model of UiO-67(Ce).[18] It contains a $[Ce_6O_4(OH)_4]^{12+}$ node and 12 monoanionic linkers. First, one bidentate linker was removed to create a vacancy and the node was capped by Ce–OH and Ce–H_2O. Then, a unit of $[Cu(OH)_2(H_2O)]$·is attached to the node.[19] The four linkers around it are benzoate (with the *p*-carbon atom kept fixed) and the rest are formate. Such approach has been successful for the simulations of metal-functionalized MOF nodes.[20,21]

The most relevant structures are shown below. They are directly compared to EXAFS data in the following sections.

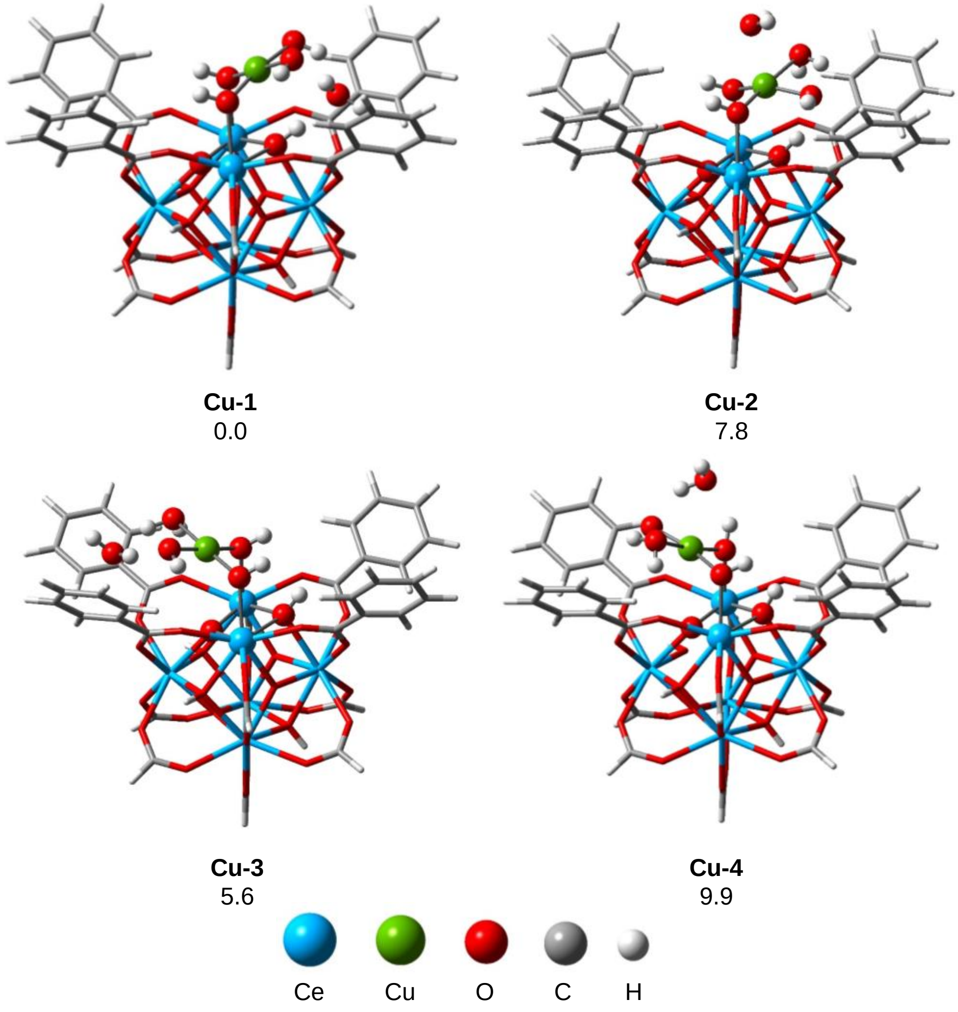

Figure S13. DFT-optimized structures of Cu^{2+} units bounded to a defective Ce-based node in Cu/UiO-67(Ce). Electronic energies with respect to **Cu-1** are shown in kcal.mol^{-1}.

Scheme S1. Cu-incorporation into a defective Ce-node. For sake of clarity the defect generation and Cu incorporation were represented separately.

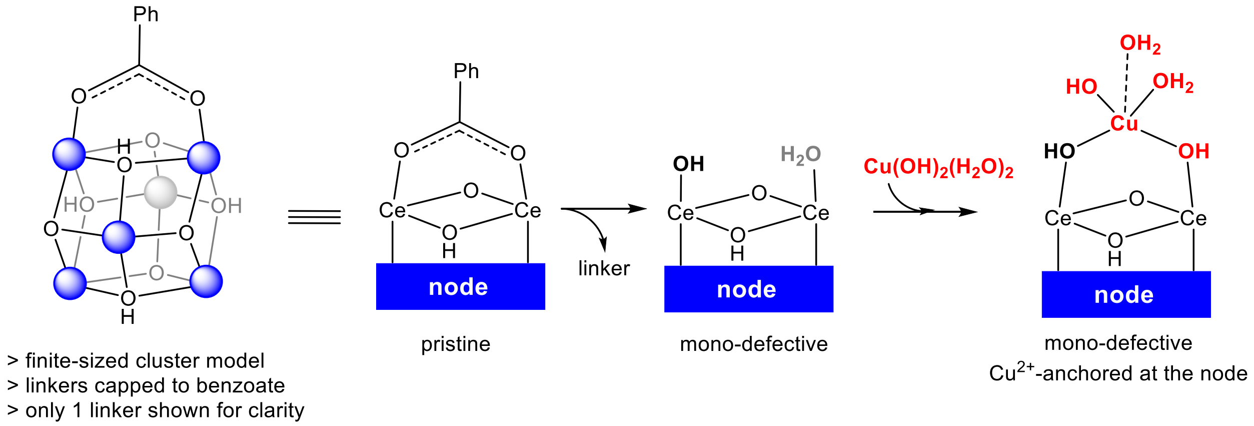

5. Operando X-Ray Absorption Spectroscopy (XAS)
5.1. Cu K-edge

Cu K-edge XANES spectra of the as-prepared sample is characterized by a pre-edge located at 8977 eV related to Cu^{2+} 1s→3d transition. The spectrum presents an intense white-line and poorly structured post-edge oscillations which are comparable with aqueous Cu^{2+}. This indicates that Cu is present in a hydrated form without an evident amount of oxide phases.

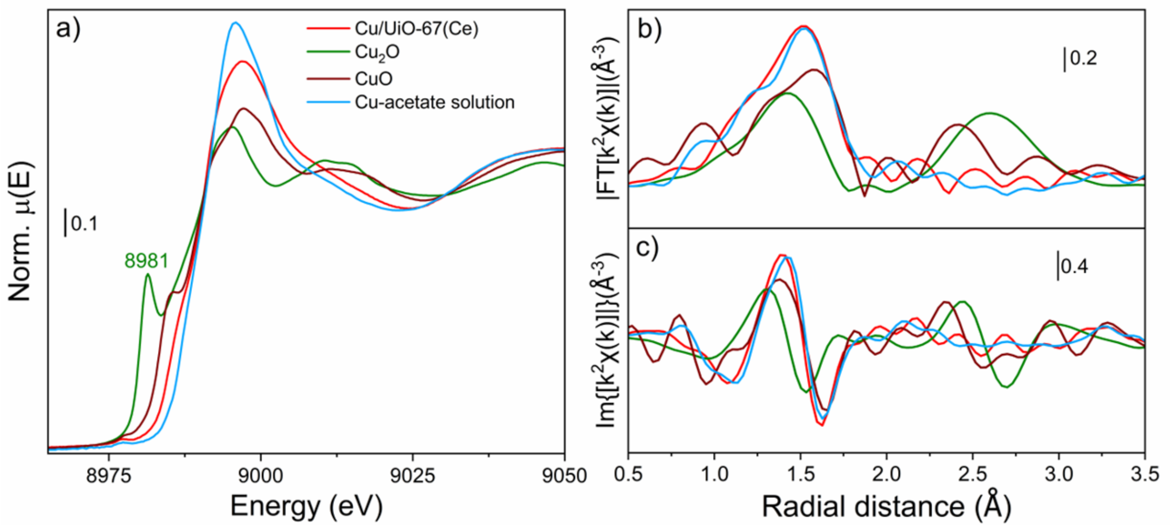

Figure S14. Cu K-edge a) XANES and k^2-weighted FT-EXAFS spectra b) magnitude and c) imaginary components of Cu/UiO-67(Ce) at RT-He (red line), CuO (dark red line), Cu_2O (green line) and Cu acetate solution (light blue line).

During thermal activation Cu K-edge XANES white-line (Figure S15a) and FT-EXAFS Cu-O shell (Figure S15b) intensities decrease in line with Cu dehydration. Coordination number of Cu-O shell during activation was not evaluated due to the presence of thermal Debye Waller contribution as observable from the dampening of EXAFS spectra intensity (Figure S15c). Formation of Cu^+ was observed during thermal activation from the ΔXANES (Figure 3b and Figure S15a inset). ΔXANES was calculated as the difference between each spectrum and the first spectrum collected at RT under He.

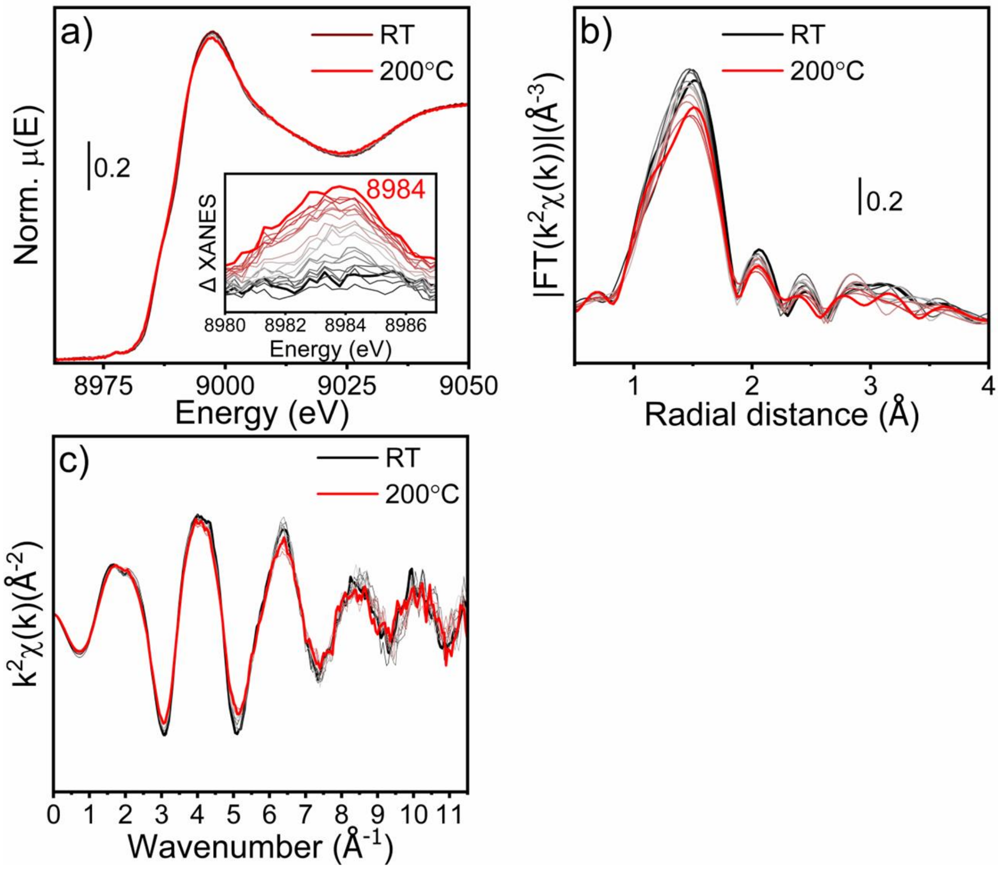

Figure S15. a) Operando Cu K-edge XANES spectra collected during Cu/UiO-67(Ce) He activation (45 mL/min) from RT (dark red line) to 200ºC (red line). b) Magnitude part of phase uncorrected k^2-weighted FT-EXAFS spectra. c) Experimental k^2-weighted EXAFS spectra.

5.2. FT-EXAFS fit procedure

5.2.1. Debye Waller factor evaluation

To investigate Cu local structure, fit of FT-EXAFS first shell was performed considering Cu-O single scattering path generate by FEFF6 using CuO as input structure. To evaluate a Cu-O coordination number (CN) without correlation with the Debye Waller factor, the fit was performed on the spectra series collected during the cooling under He conducted during the last part of the protocol. Indeed, since the catalyst was previously purged for 30’ under He, we assumed as no variation of the CN can occur. The DW factor was described with the Einstein model using the experimental temperature and an independent Einstein temperature constrained to be the same for all the datasets. The Cu-O radial distance was described considering the linear expansion model

$$R\ (Cu-O) = R_{eff} + (d_{off} + T * \alpha * R_{eff})$$

where R_{eff} is the distance from the provided atomic model while d_{off} and α are parameters refined during the fit describing the initial offset from R_{eff} and the bond expansion, respectively. ΔE and CN were fit to the same value for all the scans. The scattering amplitude factor was fixed to 0.89 after being evaluated from fit of reference Cu metal FT-EXAFS. As can be observed from the fit results in Figure S16 and Table S4 all the experimental spectra were well described by the employed model. The obtained α parameter (Table S4) indicated that no expansion occurred during cooling. However, the evaluated CN (≈3) is suspiciously low indicating as the single Cu-O scattering path might not properly describe Cu local structure.

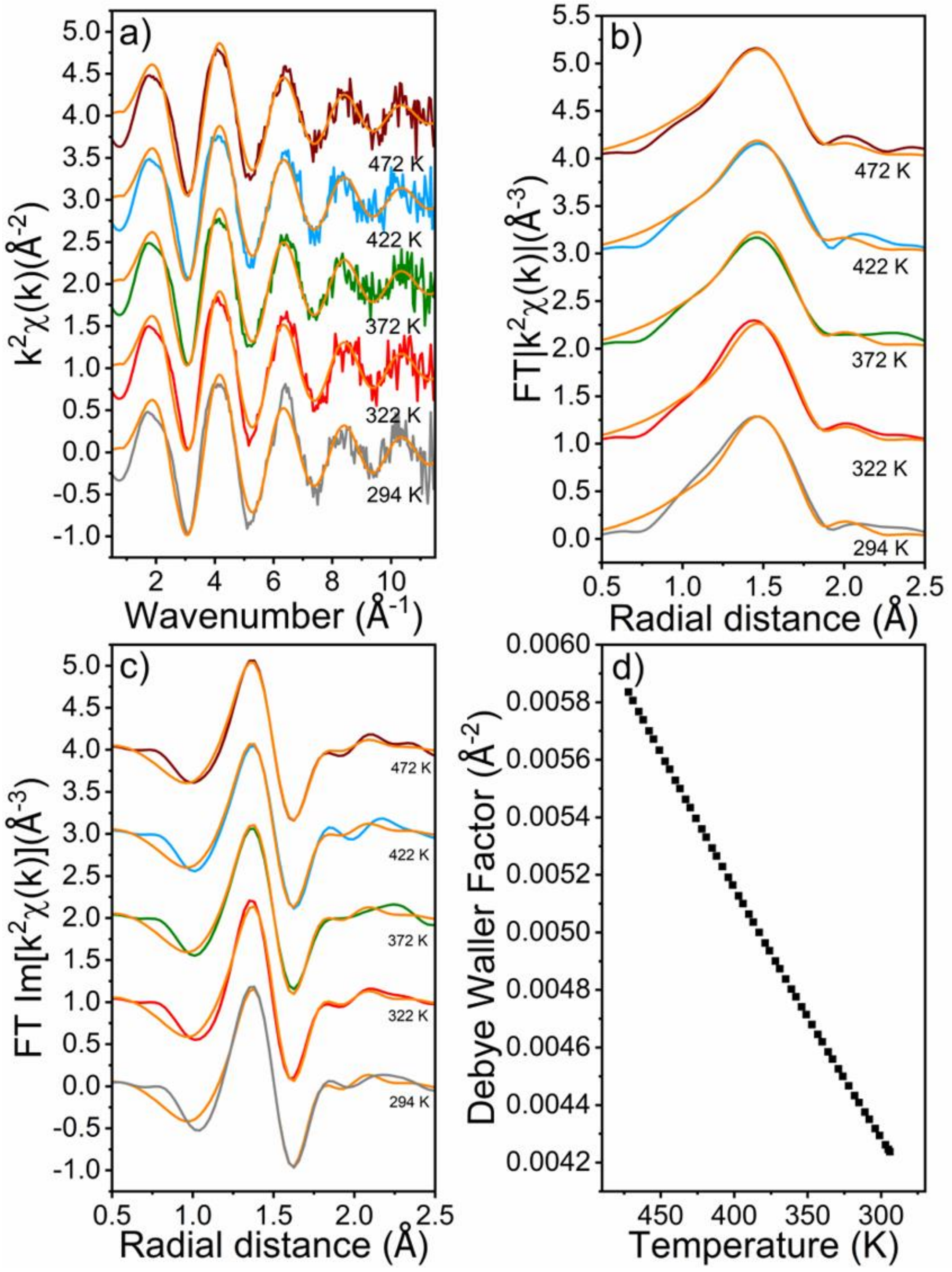

Figure S16. Experimental (colored lines) and best fit (orange line) of a) k^2-weighted EXAFS, b) phase uncorrected magnitude and c) imaginary components of k^2-weighted FT-EXAFS spectra collected during cooling from 200ºC to RT under He. For clarity, only spectra measured every 50ºC are reported. D) Cu-O DW factors (black squares) evaluated at each temperature using the Einstein model.

Table S4. Results of the fit of the FT-EXAFS collected during cooling under He. For all the spectra FT-EXAFS was extracted in the 2.2-11.4 $Å^{-1}$ k-space with Hanning window while fit was performed in the 1.4-2.4 Å range. Error bars are not reported since Larch does not evaluate errors when correlations between variables are <0.1.

Variable	Fit result
$N_{var}(N_{ind})$	65(411)
R_{eff}	1.9508 (fixed)
ΔE (eV)	2.98
d_{off} (Å)	-0.014
A (K^{-1})	2.52 e-07
CN	2.96
T_{Eins} (K)	588
R-factor	0.0096

5.2.2. EXAFS fit considering calculated structural model

The EXAFS spectra of the sample collected at RT-He was fit using three Cu-O single scattering paths calculated by Feff6 using the simulated structural model reported in Figure S13. The scattering amplitude factor was fixed to 0.89 (value obtained from fit of Cu metal foil). As reported in Figure S17c, the three Cu-O scattering paths presented imaginary components partially in antiphase, inducing a high correlation between their radial distances and DW factors. For this reason, we have constrained their DW factors to 0.0042 $Å^2$. This value was obtained from the fit during cooling reported above and it reasonably describes the DW factor value at RT. For the same reason coordination numbers were constrained to those calculated by FEFF6. With this approach the only free variable were the E_0 and the scattering paths radial distances. As reported in Table S5, all the four structures converged to the same results. For sake of clarity, the fit results are reported in Table S5 while only the best fit curves obtained using the structure **Cu-2** are reported in Figure S17.

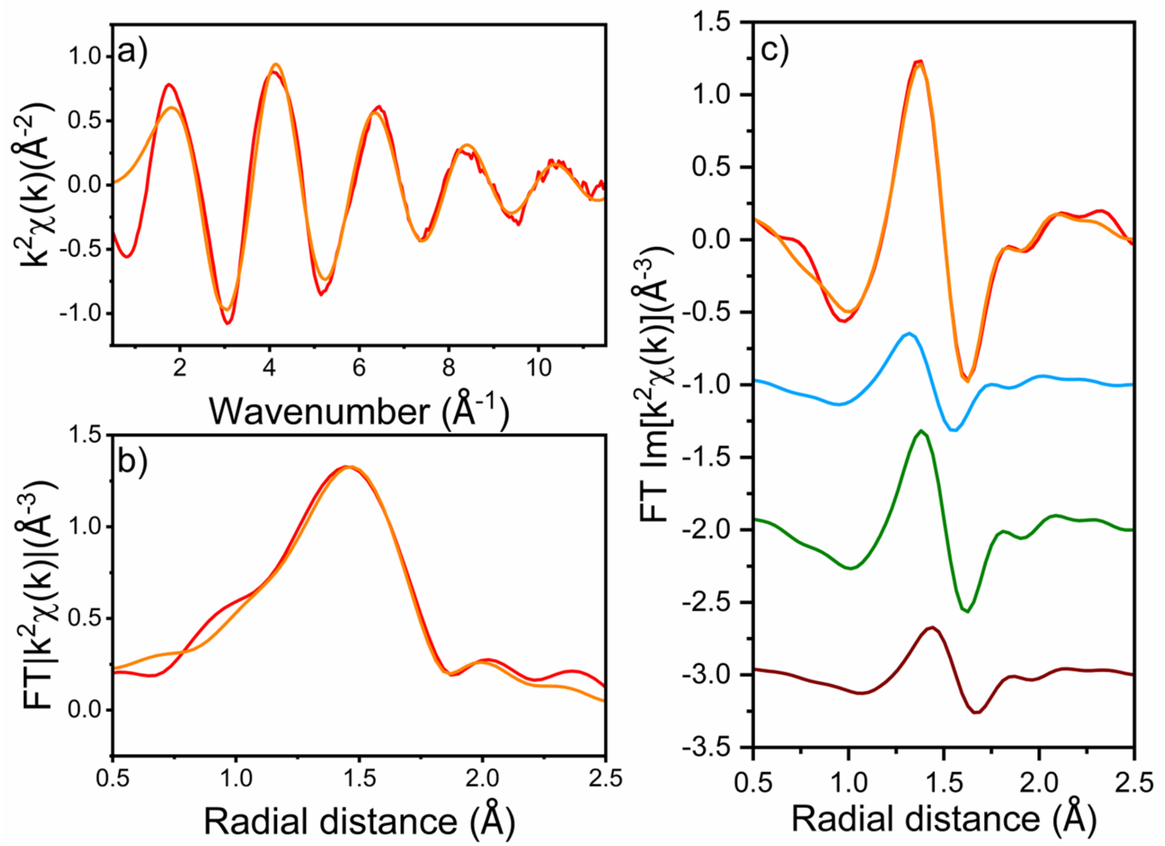

Figure S17. Experimental (red line) and best fit (orange line) of Cu/UiO-67(Ce) a) k2-weighted EXAFS and FT-EXAFS b) magnitude and c) imaginary components measured at RT under He. FT-EXAFS imaginary components of Cu-O_I, Cu-O_{II} and Cu-O_{III} are reported in light blue, green and dark red line in panel c), respectively.

Table S5. Results of the fit of the EXAFS spectra collected at RT under He after the reaction protocol. The results obtained using all the structures calculated by DFT are reported for clarity. The FT-EXAFS was extracted in the 2.2-11.4 $Å^{-1}$ k-space whit Hanning window while fit was performed in the 1.4-2.4 Å range. *These parameters were fixed and not refined.

Variable	**Cu-1**	**Cu-2**	**Cu-3**	**Cu-4**
R factor	0.0026	0.0026	0.0028	0.0026
$N_{var}(N_{ind})$	4(7)	4(7)	4(7)	4(7)
ΔE	0.5 ± 1.2	-0.5 ± 1.2	-0.5 ± 1.2	-0.5 ± 1.2
σ^2 ($Å^2$)	0.0042*	0.0042*	0.0042*	0.0042*
CN (Cu-O_I)	1*	1*	1*	1*
R-O_I (Å)[model]	1.87 ± 0.04	1.87 ± 0.04	1.87 ± 0.04	1.87 ± 0.04
CN (Cu-O_{II})	2*	2*	2*	2*
R-O_{II} (Å)[model]	1.93 ± 0.03	1.93 ± 0.03	1.93 ± 0.03	1.93 ± 0.03
CN (Cu-O_{III})	1*	1*	1*	1*
R-O_{III} (Å) [model]	1.98 ± 0.06	1.98 ± 0.06	1.98 ± 0.06	1.98 ± 0.06

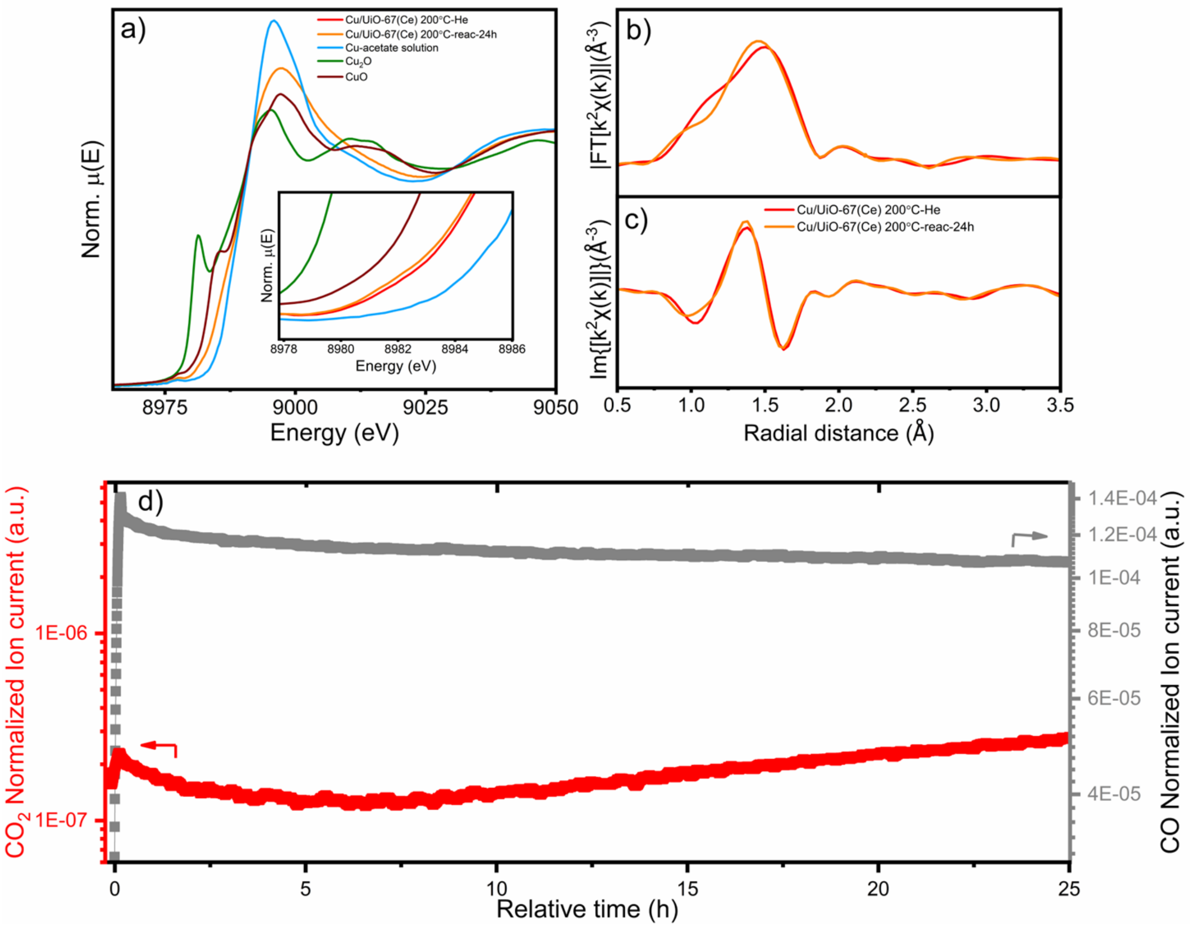

Figure S18. Cu K-edge a) XANES and k^2-weighted FT-EXAFS spectra b) magnitude and c) imaginary components of Cu/UiO-67(Ce) at 200ºC before (red line) and after exposure to CO/O_2 mixture for 24h (orange line). References CuO (dark red line), Cu_2O (green line) and Cu acetate solution (light blue line) are reported for clarity. Detail of Cu^+ 1s→4p transition is reported in the inset in panel a. d) CO and CO_2 MS signals collected during CO oxidation reaction.

5.3. Operando Ce L3-edge

Ce^{3+}/Ce^{4+} content was evaluated through Linear Combination Fit of the experimental spectra considering $Ce(NO_3)_3$ as reference for Ce^{3+}. As previously described,[22,23] instead of using CeO_2 as Ce^{4+} reference, we have employed the first spectrum of UiO-67(Ce), hypothesizing as its hydrated state contains only Ce^{4+}.

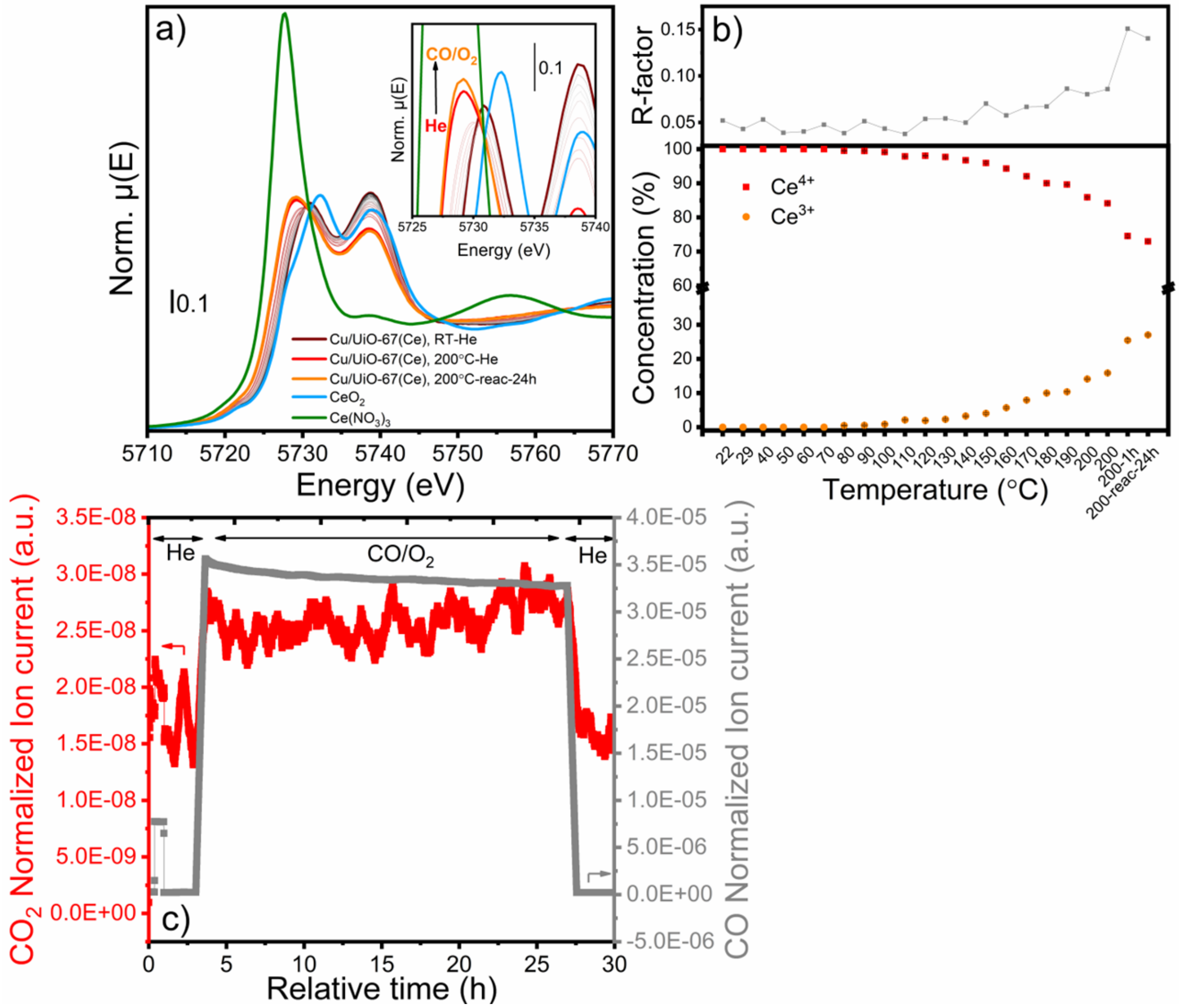

Figure S19. a) *In situ* Ce L_3-XANES spectra collected during sample activation (from RT-He to 200ºC-He, from dark red to red line). Spectrum of Cu/UiO-67(Ce) after 24h of reaction is reported with orange line while references $Ce(NO_3)_3$ and CeO_2 are reported with light blue and green lines, respectively. b) Ce^{4+}/Ce^{3+} ratio estimated from LCF. c) CO and CO_2 MS signals collected during 24h reaction.

6. References

(1) Lammert, M.; Wharmby, M. T.; Smolders, S.; Bueken, B.; Lieb, A.; Lomachenko, K. A.; De Vos, D.; Stock, N. Cerium-Based Metal Organic Frameworks with UiO-66 Architecture: Synthesis, Properties and Redox Catalytic Activity. *Chemical Communications* **2015**, *51* (63), 12578–12581.

(2) He, X.; Looker, B. G.; Dinh, K. T.; Stubbs, A. W.; Chen, T.; Meyer, R. J.; Serna, P.; Román-Leshkov, Y.; Lancaster, K. M.; Dinca, M. Cerium (IV) Enhances the Catalytic Oxidation Activity of Single-Site Cu Active Sites in MOFs. *ACS Catal* **2020**, *10* (14), 7820–7825.

(3) Thomas, S.; Marie, O.; Bazin, P.; Lietti, L.; Visconti, C. G.; Corbetta, M.; Manenti, F.; Daturi, M. Modelling a Reactor Cell for Operando IR Studies: From Qualitative to Fully Quantitative Kinetic Investigations. *Catal Today* **2017**, *283*, 176–184.

(4) Mathon, O.; Beteva, A.; Borrel, J.; Bugnazet, D.; Gatla, S.; Hino, R.; Kantor, I.; Mairs, T.; Munoz, M.; Pasternak, S. The Time-Resolved and Extreme Conditions XAS (TEXAS) Facility at the European Synchrotron Radiation Facility: The General-Purpose EXAFS Bending-Magnet Beamline BM23. *J Synchrotron Radiat* **2015**, *22* (6), 1548–1554.

(5) Newville, M. Larch: An Analysis Package for XAFS and Related Spectroscopies. In *Journal of Physics: Conference Series*; IOP Publishing, 2013; Vol. 430, p 012007.

(6) Abdel-Mageed, A. M.; Rungtaweevoranit, B.; Parlinska-Wojtan, M.; Pei, X.; Yaghi, O. M.; Behm, R. J. Highly Active and Stable Single-Atom Cu Catalysts Supported by a Metal–Organic Framework. *J Am Chem Soc* **2019**, *141* (13), 5201–5210. https://doi.org/10.1021/jacs.8b11386.

(7) Abdel-Mageed, A. M.; Rungtaweevoranit, B.; Impeng, S.; Bansmann, J.; Rabeah, J.; Chen, S.; Häring, T.; Namuangrak, S.; Faungnawakij, K.; Brückner, A. Unveiling the CO Oxidation Mechanism over a Molecularly Defined Copper Single-Atom Catalyst Supported on a Metal–Organic Framework. *Angewandte Chemie International Edition* **2023**, *62* (30), e202301920.

(8) Elias, J. S.; Artrith, N.; Bugnet, M.; Giordano, L.; Botton, G. A.; Kolpak, A. M.; Shao-Horn, Y. Elucidating the Nature of the Active Phase in Copper/Ceria Catalysts for CO Oxidation. *ACS Catal* **2016**, *6* (3), 1675–1679. https://doi.org/10.1021/acscatal.5b02666.

(9) Wang, B.; Zhang, H.; Xu, W.; Li, X.; Wang, W.; Zhang, L.; Li, Y.; Peng, Z.; Yang, F.; Liu, Z. Nature of Active Sites on Cu–CeO2 Catalysts Activated by High-Temperature Thermal Aging. *ACS Catal* **2020**, *10* (21), 12385–12392. https://doi.org/10.1021/acscatal.0c03188.

(10) Zhao, Y.; Truhlar, D. G. A New Local Density Functional for Main-Group Thermochemistry, Transition Metal Bonding, Thermochemical Kinetics, and Noncovalent Interactions. *J Chem Phys* **2006**, *125* (19).

(11) Frisch, M. J. ea; Trucks, G. W.; Schlegel, H. B.; Scuseria, G. E.; Robb, M. A.; Cheeseman, J. R.; Scalmani, G.; Barone, V.; Petersson, G. A.; Nakatsuji, H. Gaussian 16, Revision C. 01. **2016**.

(12) Andrae, D.; Häußermann, U.; Dolg, M.; Stoll, H.; Preuß, H. Energy-Adjustedab Initio Pseudopotentials for the Second and Third Row Transition Elements. *Theor Chim Acta* **1990**, *77* (2), 123–141. https://doi.org/10.1007/BF01114537.

(13) Hariharan, P. C.; Pople, J. A. The Influence of Polarization Functions on Molecular Orbital Hydrogenation Energies. *Theor Chim Acta* **1973**, *28* (3), 213–222. https://doi.org/10.1007/BF00533485.

(14) Ditchfield, R.; Hehre, W. J.; Pople, J. A. Self-Consistent Molecular-Orbital Methods. IX. An Extended Gaussian-Type Basis for Molecular-Orbital Studies of Organic Molecules. *J Chem Phys* **2003**, *54* (2), 724–728. https://doi.org/10.1063/1.1674902.

(15) Hehre, W. J.; Ditchfield, R.; Pople, J. A. Self—Consistent Molecular Orbital Methods. XII. Further Extensions of Gaussian—Type Basis Sets for Use in Molecular Orbital Studies of Organic Molecules. *J Chem Phys* **2003**, *56* (5), 2257–2261. https://doi.org/10.1063/1.1677527.

(16) Álvarez-Moreno, M.; de Graaf, C.; López, N.; Maseras, F.; Poblet, J. M.; Bo, C. Managing the Computational Chemistry Big Data Problem: The IoChem-BD Platform. *J Chem Inf Model* **2015**, *55* (1), 95–103. https://doi.org/10.1021/ci500593j.

(17) *http://dx.doi.org/10.19061/iochem-bd-6-322*.

(18) Wu, X.-P.; Gagliardi, L.; Truhlar, D. G. Cerium Metal–Organic Framework for Photocatalysis. *J Am Chem Soc* **2018**, *140* (25), 7904–7912. https://doi.org/10.1021/jacs.8b03613.

(19) Ikuno, T.; Zheng, J.; Vjunov, A.; Sanchez-Sanchez, M.; Ortuño, M. A.; Pahls, D. R.; Fulton, J. L.; Camaioni, D. M.; Li, Z.; Ray, D.; Mehdi, B. L.; Browning, N. D.; Farha, O. K.; Hupp, J. T.; Cramer, C. J.; Gagliardi, L.; Lercher, J. A. Methane Oxidation to Methanol Catalyzed by Cu-Oxo Clusters Stabilized in NU-1000 Metal–Organic Framework. *J Am Chem Soc* **2017**, *139* (30), 10294–10301. https://doi.org/10.1021/jacs.7b02936.

(20) Bernales, V.; Ortuño, M. A.; Truhlar, D. G.; Cramer, C. J.; Gagliardi, L. Computational Design of Functionalized Metal–Organic Framework Nodes for Catalysis. *ACS Cent Sci* **2018**, *4* (1), 5–19. https://doi.org/10.1021/acscentsci.7b00500.

(21) Yousuf, M. R.; Johnson, E. M.; Maynes, A. J.; Johnston, C. R.; Karim, A. M.; Morris, A. J.; Morris, J. R.; Troya, D. Catalytic CO Oxidation by Cu Single Atoms on the UiO-66 Metal–Organic Framework: The Role of the Oxidation State. *The Journal of Physical Chemistry C* **2022**, *126* (30), 12507–12518. https://doi.org/10.1021/acs.jpcc.2c03463.

(22) Smolders, S.; Lomachenko, K. A.; Bueken, B.; Struyf, A.; Bugaev, A. L.; Atzori, C.; Stock, N.; Lamberti, C.; Roeffaers, M. B. J.; De Vos, D. E. Unravelling the Redox-Catalytic Behavior of Ce4+ Metal–Organic Frameworks by X-Ray Absorption Spectroscopy. *ChemPhysChem* **2018**, *19* (4), 373–378. https://doi.org/https://doi.org/10.1002/cphc.201700967.

(23) Rojas-Buzo, S.; Salusso, D.; Bonino, F.; Paganini, M. C.; Bordiga, S. Unraveling the Reversible Formation of Defective Ce3+ Sites in the UiO-66 (Ce) Material: A Multi-Technique Study. *Mater Today Chem* **2023**, *27*, 101337.
